# Energizing effects of bupropion on effortful behaviors in mice under positive and negative test conditions: modulation of DARPP-32 phosphorylation patterns

**DOI:** 10.1007/s00213-021-05950-4

**Published:** 2021-09-09

**Authors:** Carla Carratalá-Ros, Régulo Olivares-García, Andrea Martínez-Verdú, Edgar Arias-Sandoval, John D. Salamone, Mercè Correa

**Affiliations:** 1 Àrea de Psicobiologia, Universitat Jaume I, Campus de Riu Sec, 12071 Castelló, Spain; 2 Behavioral Neuroscience Division, University of Connecticut, Storrs, CT 06269-1020 USA

**Keywords:** Bupropion, Dopamine, Motivation, Anergia, Fatigue, Effort, Depression, Nucleus accumbens

## Abstract

Motivational symptoms such as anergia, fatigue, and reduced exertion of effort are seen in depressed people. To model this, nucleus accumbens (Nacb) dopamine (DA) depletions are used to induce a low-effort bias in rodents tested on effort-based decision-making. We evaluated the effect of the catecholamine uptake blocker bupropion on its own, and after administration of tetrabenazine (TBZ), which blocks vesicular storage, depletes DA, and induces depressive symptoms in humans. Male CD1 mice were tested on a 3-choice-T-maze task that assessed preference between a reinforcer involving voluntary physical activity (running wheel, RW) vs. sedentary activities (sweet food pellet intake or a neutral non-social odor). Mice also were tested on the forced swim test (FST), two anxiety-related measures (dark–light box (DL), and elevated plus maze (EPM)). Expression of phosphorylated DARPP-32 (Thr34 and Thr75) was evaluated by immunohistochemistry as a marker of DA-related signal transduction. Bupropion increased selection of RW activity on the T-maze. TBZ reduced time running, but increased time-consuming sucrose, indicating an induction of a low-effort bias, but not an effect on primary sucrose motivation. In the FST, bupropion reduced immobility, increasing swimming and climbing, and TBZ produced the opposite effects. Bupropion reversed the effects of TBZ on the T-maze and the FST, and also on pDARPP32-Thr34 expression in Nacb core. None of these manipulations affected anxiety-related parameters. Thus, bupropion improved active behaviors, which were negatively motivated in the FST, and active behaviors that were positively motivated in the T-maze task, which has implications for using catecholamine uptake inhibitors for treating anergia and fatigue-like symptoms.

## Introduction

Psychiatric pathologies such as depression are characterized not only by emotional and cognitive symptoms, but also by motivational deficiencies that lead to manifestations such as anergia, psychomotor slowing, fatigue, and lassitude (Stahl 2002; Demyttenaere et al. 2005; Treadway and Zald 2011; Fava et al. 2014; Salamone et al. 2015; Barch et al. 2016). The severity of these symptoms limits long-term functional outcomes, producing problems with employment and social functions (Salamone et al. 2007; Fava et al. 2014; Rothschild et al. 2014; Chong et al. 2016). People with major depression have deficits in the exertion of effort, reward seeking, and effort-related decision-making that are not necessarily dependent upon alterations in the experience of pleasure in response to primary stimuli (Treadway and Zald 2011; Argyropoulos and Nutt 2013; Pizzagalli 2014; Knowland and Lim 2018).

Several lines of evidence point to the involvement of the mesolimbic dopamine (DA) system, particularly the DA innervation of nucleus accumbens (Nacb), in effort-related dysfunctions potentially related to depressive symptoms (Caligiuri and Ellwanger 2000; Schmidt et al. 2001; Volkow et al. 2001; Salamone et al. 2007, 2016, 2018). In animal models, the administration of the vesicular monoamine transporter type-2 (VMAT-2) blocker and DA-depleting agent tetrabenazine (TBZ) produces effort-related motivational dysfunction, characterized by shifts in behavior from physically demanding options that lead to highly preferred reinforcers, towards low-effort ones that lead to less valued reinforcers (Carratalá-Ros et al. 2020, 2021; Correa et al. 2018; López-Cruz et al. 2018; Nunes et al. 2013; Pardo et al. 2015; Randall et al. 2012; Rotolo et al. 2019, 2020; Yang et al. 2020a, b; Yohn et al. 2016b, c). TBZ in humans has been used for the treatment of Huntington disease, and it has been reported to induce depressive symptoms mainly related to fatigue and psychomotor retardation (Frank 2009; Guay 2010).

Moreover, it has been shown that blockade of the DA transporter (DAT), which leads to increases in extracellular DA, can improve motivational symptoms in depressed patients (Patkar et al. 2006; Goss et al. 2013; Malhi et al. 2016). Thus, the catecholamine uptake inhibitor bupropion has demonstrated efficacy to treat motivational symptoms in depression (Feighner et al. 1986; Kiev et al. 1994; Weihs et al. 2000; Papakostas 2006; Pae et al. 2007; Cooper et al. 2014). Its main mechanism of action is to block the catecholamine transporters DAT and NET (norepinephrine transporter), and it shows low potential to produce abuse in humans (Learned-Coughlin et al. 2003; Stahl et al. 2004; Nutt et al. 2007) and in rodents (Mori et al. 2013). In addition, it has been shown that bupropion increases extracellular DA and DA-related signal transduction in Nacb (Randall et al. 2014, 2015). Considerable evidence indicates that enhancement of DA transmission can reverse effort-decision-making impairments (Salamone et al. 2007, 2015, 2016; Mai et al. 2012; Hosking et al. 2015; Yohn et al. 2015a, 2016b, c, d, e; Floresco et al. 2018; Rotolo et al. 2019, 2020). Thus, in operant tasks (progressive (PROG) or fixed ratio (FR5) vs chow free feeding), bupropion was able to alleviate and reverse the effects produced by TBZ, increasing the effort to work in order to obtain a preferred food (Randall et al. 2014; Yohn et al. 2015a, 2015b, 2016b).

In evaluating effort-based decision-making processes in rodent models, lever pressing is the most commonly used work requirement (Treadway et al. 2012; Randall et al. 2014; Yohn et al. 2015a, b, 2016b, c). However, more whole-body physical activities such as rearing (Yang et al. 2020a, b), barrier climbing (Salamone 1994), or wheel running can be used as the requirement to get access to the most preferred reinforcer, or even as the more preferred activity (Correa et al. 2016, 2020). Physical activities can require high levels of performance and endurance, but they also can have intrinsic motivational properties (Belke 2006; Belke and Pierce 2014). One of the most commonly studied voluntary physical activities in rodent models is wheel running, which can be performed under non-stressed conditions. Mice run spontaneously when given access to running wheels, and, depending on the strain, they can run for a total distance of up to 20 km per day and a total activity time of around 3 to 7 h a day (Manzanares et al. 2019). Thus, wheel running can be used as the highly preferred and highly demanding choice in relation to other less preferred but less effort demanding alternatives, such as consumption of palatable food or drugs of abuse (Premack and Premack 1963; McMillan et al. 1995; Mueller et al. 1997; Cosgrove et al. 2002; Correa et al. 2016, 2020; López-Cruz et al. 2018; Carratalá-Ros et al. 2020; Presby et al. 2020). However, little is known about the neural mechanisms involved in the decision-making processes that establish those preferences for vigorous physically demanding behaviors.

Therefore, the present study explored the impact of bupropion, administered alone or in combination with the DA-depleting agent TBZ, on performance in the 3-choice T-maze task, which uses voluntary exercise in a running wheel as the most preferred but high effort demanding option in competition with less preferred but more sedentary options such as consumption of high-sucrose–containing food or sniffing floral odors (Carratalá-Ros et al. 2020; Correa et al. 2020; López-Cruz et al. 2018). Because stress can biphasically affect how much animals run: acute stress increases short-term running (Gurfein et al. 2012), while mild chronic stress reduces total spontaneous wheel running in mice (DeVallance et al. 2017), we compared the impact of bupropion alone or after TBZ administration, on spontaneous preference under non-stressful conditions (animals were non-food restricted, running was voluntary, and the smell used had demonstrated to be neutral for mice in previous studies (López-Cruz et al. 2018).

In addition, because aversive states such as stress and anxiety play a role in depression and could affect the activational component of motivated behaviors (Shafiei et al. 2012), we assess if the present pharmacological manipulations have an impact on the forced swim test (FST). This paradigm presents a stressful and non-escapable experimental setting such a deep tank full of water in which naïve rodents at first try to actively scape, but after a while, they give up and remain immobile (mainly floating) or mildly swimming to stay afloat (Porsolt et al. 1977; Armario and Nadal 2013). This immobility in the FST can be reversed by different categories of drugs, including drugs used as antidepressants (Porsolt et al. 1977; Lucki 1997; Gil and Armario 1998). However, vigorous escape-related behaviors such as climbing can be also modified by antidepressant drugs (Armario et al 1988; Lucki 1997). Furthermore, there is evidence that active behaviors are more likely to be affected by DA manipulations than the traditional immobility measure (Gil and Armario 1998; Costa et al. 2013). Thus, TBZ increases immobility in mice and rats (Jang et al. 2009; Wang et al. 2010; Carratalá-Ros et al. 2020, 2021), but also has shown to dose dependently produced a significant decrease in climbing (Carratalá-Ros et al. 2020), and bupropion has antiimmobility effects in mice and rats (Yamada et al. 2004; Kitamura et al. 2010; Yuen et al. 2017), but also increases swimming and climbing (Rénéric and Lucki 1998; Hayashi et al. 2011; Yuen et al. 2017). Moreover, in the same animals, we evaluated anxiety-related behaviors in the dark and light box (DL) and the elevated plus maze (EPM).

Finally, an additional experiment studied how the most effective dose of bupropion on these behaviors would affect DA receptor–dependent metabotropic markers in Nacb using immunochemistry and ventral striatum using Western blotting methods.

## Materials and methods

### Animals

CD1 adult male mice (*N* = 148) purchased from Janvier, France S.A. were 8–14 weeks old (40–50 g) at the beginning of the study. Mice were housed in groups of three or four per cage, with standard laboratory rodent chow and tap water available ad libitum. The colony was kept at a temperature of 22 ± 2 °C with lights on from 08:00 to 20:00 h. All animals were under a protocol approved by the Institutional Animal Care and Use committee of Universitat Jaume I. All experimental procedures complied with directive 2010/63/EU of the European Parliament and of the Council, and with the “Guidelines for the Care and Use of Mammals in Neuroscience and Behavioral Research”, National Research Council 2003, USA. All efforts were made to minimize animal suffering and to reduce the number of animals used.

### Pharmacological agents

The catecholamine uptake inhibitor bupropion hydrochloride (Alfa Aesar, Spain) was dissolved in 0.9% saline, which also served as the vehicle control for these studies. Bupropion was administered 30 min before tests started. Specific doses and times were selected from previous work in our laboratory using effort based choice decision-making tasks (Nunes et al. 2013; Randall et al. 2014; Yohn et al. 2015b) and from studies with the forced swim test (Yamada et al. 2004; Kitamura et al. 2010). The VMAT-2 inhibitor tetrabenazine (TBZ, CIMYT Quimica SL, Spain) was dissolved in a solution of 0.9% saline (80%) plus dimethylsulfoxide (DMSO 20%, final pH 5.5) and administered 120 min before testing. The vehicle solution of 20% DMSO and saline was used as the vehicle control for TBZ. Time and doses were selected based on previous work (Correa et al. 2018; López-Cruz et al. 2018; Carratalá-Ros et al. 2020). All drugs were administered intraperitoneally (IP).

### Testing procedures

All behavioral procedures started 2 h after light period onset. The behavioral test room was illuminated with a soft light, and external noise was attenuated.

#### T-maze RW-sucrose-odor choice task

**Fig. 1:**
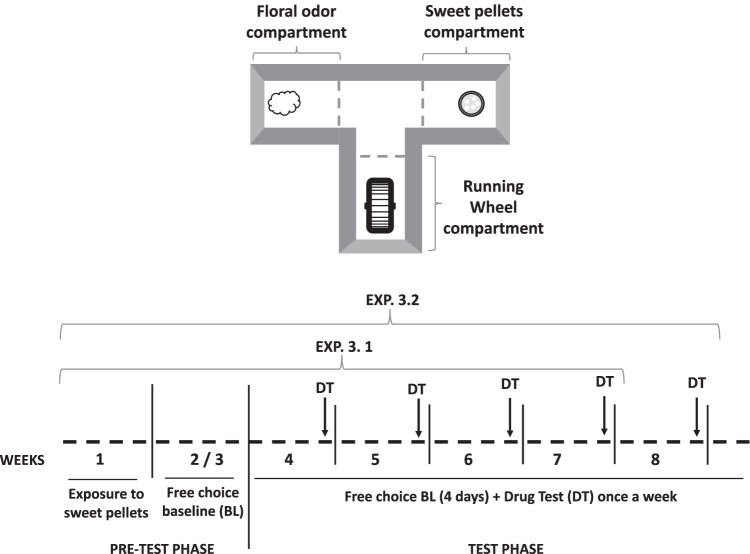
Schematic representation of the three-choice T-maze task settings and timeline for the different experimental phases for the tetrabenazine (TBZ), bupropion (BUP), and TBZ-BUP experiments

The T-maze apparatus (25 cm L × 11 cm W × 30 cm H) consisted of a central area that lead to 3 arms (based on Correa et al. 2020, 2016; see Fig. 1). Each arm provided different types of stimuli. In one of them, sweet pellets (TestDietTM, 50% sucrose, 45 mg each) were available; in another arm, there was a RW, and in the third arm, there was a hole with a cotton ball socked with a neutral non-social odor (López-Cruz et al. 2018; Carratalá-Ros et al. 2020). Training as well as test sessions lasted 15 min. Mice were trained 5 days a week. In training phase 1, to avoid neophobia to the sweet tasting pellets, animals were enclosed in that arm with the food during 5 sessions. In training phase 2, during 2 more weeks, animals were given one 15-min session a day to the T-maze with free access to the three stimuli. Test phase lasted during 4 or 5 more weeks depending on the experiment. For each week, there were 4 baseline days plus a testing session in which animals received drug injections. Test sessions were videotaped and a trained observer unaware of the experimental condition manually registered several parameters. Time interacting with each of the stimuli was selected as the main dependent measure because it allowed for the evaluation of the three stimuli with the same scale. Time allocation is a useful measure of preference, relative reinforcement value, and response choice (Baum and Rachlin 1969). Entries into the arms (as a measure of exploration) and time spent in the arms of the T-maze (as an index confirming proximity to the stimuli) were simultaneously recorded. All these measures were taken based on previous studies (Correa et al. 2016, 2020; López-Cruz et al. 2018; Carratalá-Ros et al. 2020).

#### Forced swim test

This paradigm has been used as a test for evaluating drugs with potential as antidepressants (Porsolt et al. 1977). Immobility is the main dependent variable evaluated, and was defined as the period during which the animal remained motionless, making only minor movements to balance the body and keep the head above the water. This immobility response has been considered as a measure for depression and despair which is a very anthropomorphical way of understanding a switch from active to passive behavior in the face of an acute stressor that, in fact, is behaviorally adaptive and reveals coping strategies in an inescapable situation (Molendijk and de Kloet 2015; Armario 2021). Thus, in the present group of experiments, active behaviors were also recorded. Swimming was recorded when animals carried out horizontal movements with their forepaws, leading to the displacement of the body throughout the swim chamber. In addition, we also assessed escape-related mobility behavior such climbing (Armario et al. 1988; Gil and Armario 1998). Climbing is defined as any energetic and vertical movement of all four limbs against the wall of the tank. Naïve mice were placed in a transparent cylindrical glass tank (26-cm high and 18-cm diameter) filled with water (14 cm) and maintained at a temperature of 25 °C. Water was changed between animals. During the 6-min test, mice were videotaped from the side, and climbing, immobility, and mild swimming were later measured by an observer unaware of the experimental condition. After the test, mice were dried with a soft towel, put back in a box with absorbing paper under a warming light, and were monitored for 10 min.

Because this same animals received 7 days later the same doses and drug combinations and were evaluated in the anxiety tests, we performed correlational analysis to observe potential pathological traits (Armario 2021).

#### Dark and light box

The DL test is based on the conflict between the tendency to explore a novel environment and the avoidance of a brightly lighted open area (Blumstein and Crawley 1983). The DL apparatus consisted of a polypropylene chamber divided in two compartments by a partition containing a small opening (5 cm H × 5 cm W). The light compartment (25 cm W × 25 cm H × 25 cm L) was open, painted in white, and illuminated (335 lx), while the dark compartment (25 cm W × 25 cm H × 18 cm L) was painted in black and had a removable ceiling to close it. To start the test session, mice were individually placed in the dark chamber facing one corner. Test sessions were videotaped, and the latency of the first entry into the lit chamber, the total number of crosses, and the total time spent in the lit chamber were recorded for 5 min (procedure is based on López-Cruz et al. 2017).

#### Elevated plus maze

After being in the DL box for 5 min, animals were placed in the EPM for 5 more minutes. The EPM consists of two open and two enclosed arms (65 cm L × 5 cm W) arranged in a plus configuration and intersecting in a central platform. It is made of black polypropylene and is elevated 50 cm above the floor. The open arms have a 1-cm border around their perimeter, and the closed arms have a 20-cm translucent wall. This anxiety paradigm measures the avoidance that rodents show to elevated open spaces. Under normal conditions, mice spent more time and make more entries into the closed arms of the maze (Lister 1987). Animals were placed in the central platform with their head pointing at one enclosed arm, and they were assessed during 5 min. Sessions were videotaped, and a trained observer unaware of the experimental condition registered total time spent in the open arms and also total number of entries in the four arms as an index of locomotion. An entry into an arm was recorded when the animal crossed with all four legs the line that connected that arm with the central platform (procedure is based on López-Cruz et al. 2017).

### Immunohistochemistry for phosphorylated DA and c-AMP-related phosphoprotein-32 kDaltons

After drug treatments, animals were anesthetized with CO2 and transcardially perfused with 0.9% physiological saline for 5 min, followed by perfusion with 3.7% formaldehyde for 5 min, and then brains were extracted. Tissue was fixed in 3.7% formaldehyde overnight and moved to 30% sucrose cryo-protectant. Then 40-um sections were cut using a cryostat. To measure the immunoreactivity to phosphorylated DA and c-AMP-regulated phosphoprotein 32 kDa (pDARPP-32), nonspecific binding sites were blocked with a solution of 3% H202 for 30 min at room temperature following then with 1% bovine serum albumin and 0.1% Triton X-100 in PBS for 1 h at room temperature. Phosphorylated DARPP-32 immunoreactivity was visualized with a polyclonal rabbit antibody for pDARPP-32 phosphorylated at threonine 34 residue (Thr34, 1:1000; Santa Cruz Biotechnology) or polyclonal rabbit antibody for pDARPP-32 phosphorylated at threonine 75 residue (Thr75, 1:500, Santa Cruz Biotechnology). These antibodies were dissolved in solutions that also contained 0.1% bovine serum albumin and 0.1 Triton X-100 in PBS for 24 h incubation on a rotating shaker at 4 °C. After the primary antibody treatment, the sections were rinsed in PBS (3 times for 5 min) and incubated in the secondary antibody, anti-rabbit HRP conjugate envision plus (DAKO) for 1 h and 30 min on a rotating shaker at room temperature. Finally, sections were washed and rinsed for 3–5 min in 3,3’ diaminobenzidine chromogen. The sections were then mounted to gelatin-coated slides, air dried, and coverslipped using Eukitt quick-hardening (Sigma-Aldrich) as a mounting medium. The tissue was then examined by light microscopy.

Quantification of the number of cells that express immunoreactivity for pDARPP-32 (Thr34) and pDARPP-32 (Thr75) in the Nacb core was performed by photographing the sections with a 20 × (0.125 mm^2^/field) objective (Eclipse E600; Nikon) upright microscope equipped with a Leica DFC 450C camera (Leica Microsystems) and captured digitally using LASX software. Cells that were positively labeled for pDARPP-32 (Thr34) and pDARPP-32 (Thr75) were quantified with ImageJ software (version 1.51) and a macro written to automate particle counting within the region of interest. The size of the region of interest was 1000 × 1000 um. For each animal, cell counts were at levels that correspond to 0.70–1.70 mm anterior to bregma (Paxinos and Franklin 2004) bilaterally from at least three sections and counts were averaged across slides and sections.

### Western blotting for phosphorylated DA and c-AMP related phosphoprotein-32 kDaltons

We further analyzed phosphorylation of DARPP-32 at the Thr75 using an additional biochemical technique and a different primary antibody for pDARPP-32(Thr75) to exclude lack of sensitivity of the method as the cause of the negative immunohistochemical results. After drug treatments, animals were anesthetized with CO_2_ and were decapitated, and brains were extracted. Fresh striatal tissue samples were homogenized in ice-cold lysis buffer (130 Mm NaCl, 20 Mm Tris–Hcl at Ph 8.0, and 1% Nonidet P40), containing protease inhibitors (10 ug/ml of aprotinin, 20ug/ml of leupetine, and 1 Mm PMSF) as well as phosphatase inhibitors (10 Mm NaF, 1 Mm sodium orthovanade and 10 Mm DTT). Homogenates were centrifuged at 13,000 rpm for 15 min at 4 °C. Aliquots of supernatants were collected and used Bradford quantification of total protein. Every sample was boiled for 3 min for protein denaturation. Equal amounts (30ug) of striatum protein samples were separated by 12.5% SDS-PAGE and transferred to nitrocellulose membrane (Bio-rad) for 90 min at 30 V. Filtering membranes were incubated in 5% of bovine serum albumin (BSA, Sigma-Aldrich) dissolved in TBS-T (Tris Base 200 Mm and 5 M NaCl) containing 0.1% Tween 20, and then incubated overnight at 4 °C with polyclonal rabbit anti-DARPP32-Thr75 antibody (1:500, cell signaling). Membranes were probed with anti-actin polyclonal antibody (1:500, Abcam), as an internal standard for protein quantification. After rinses with TBS-T buffer, membranes were incubated with goat anti-rabbit IG secondary antibody coupled to HRP (1:20,000, Bio-rad) during 1 h and developed by enhanced chemiluminescence system (1:40, ThermoFisher Scientific). The membranes were exposed to Image Quant LAS 4000 (Leica). The relative densities of brands were analyzed using ImageJ software. Every sample was replicated at least twice to ensure the reproducibility of the method.

### Statistical analyses

Normally distributed data (according to Kolmogorov-Smirnov test) in the T-maze experiment followed a within groups design. Thus, data were analyzed by repeated measures ANOVA. Normally distributed and homogenous data for the FST, DL, EPM, and the biochemical experiments employed a between groups design, and data were analyzed by one-way ANOVA. When the overall ANOVA was significant, non-orthogonal planned comparisons using the overall error term were used to compare each treatment with the vehicle control group (Keppel 1991). For these comparisons, α level was kept at 0.05 because the number of comparisons was restricted to the number of treatments minus one. To evaluate the relation between the behaviors in the FST with the behaviors in the DL and EPM paradigms done in the same mice, Pearson r correlational coefficient was used. All data were expressed as mean ± SEM, and significance was set at *p* < 0.05. STATISTICA 7.0 software was used.

## Results

### Effect of bupropion on preference for reinforcers as measured in the 3-choice-T-maze task

**Fig. 2:**
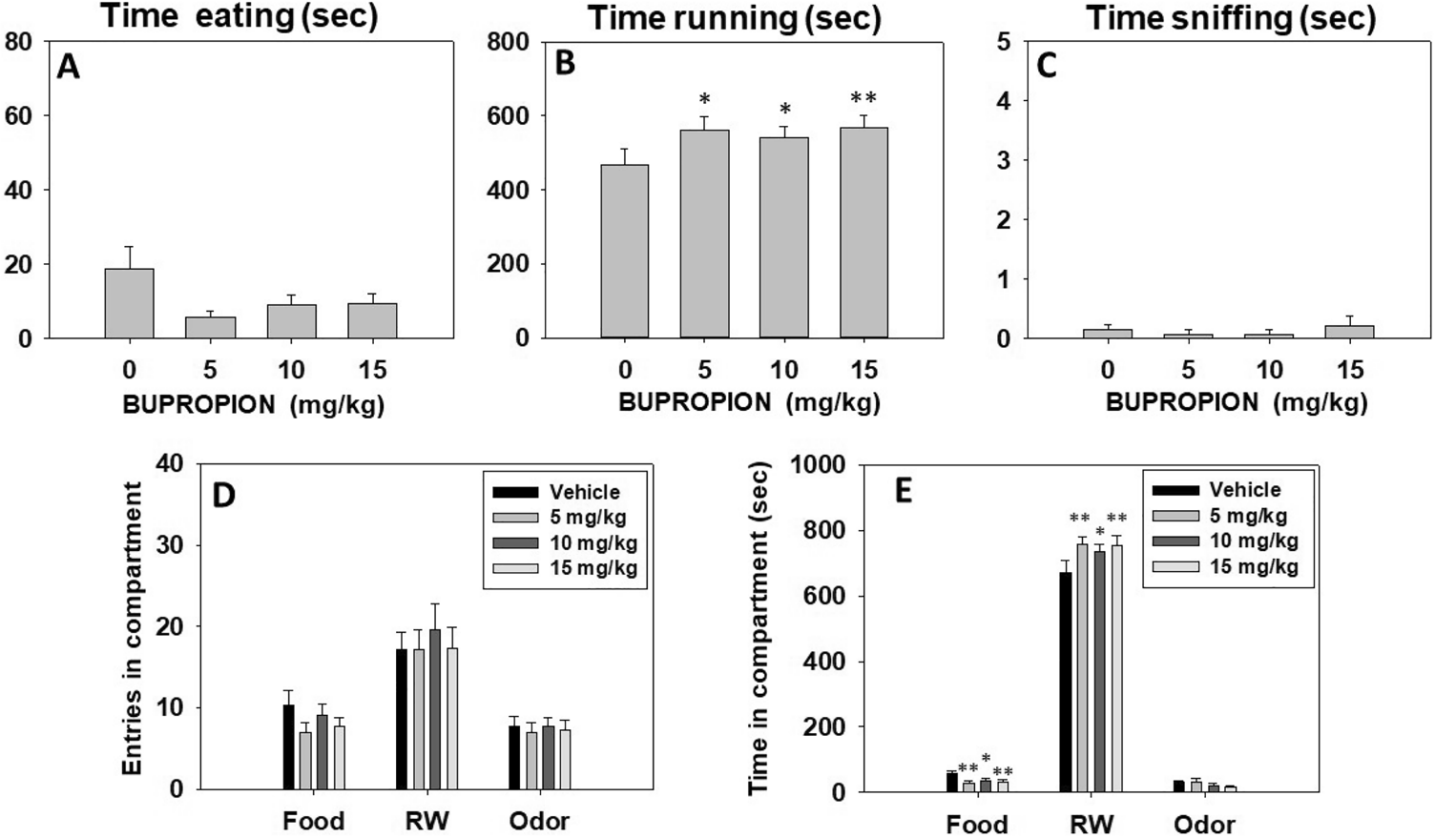
Effect of bupropion (vehicle, 5, 10, and 15 mg/kg) on time eating (**A**), time running (**B**), time sniffing (**C**), entries into compartments (**D**), and time spent in each compartment (**E**) of the T-maze task assessed during 15 min. Bars represent mean ± S.E.M. of accumulated seconds or number of entries. *p < 0.05, **p < 0.01 significantly different from vehicle

Animals (*N* = 14) received a dose of BUP (0.0, 5.0, 10.0, and 15.0 mg/kg) once a week in a randomly varied order. The T-maze paradigm requires a baseline performance of two weeks before tests start, and that performance was maintained across weeks, thus allowing for a repeated measures design. Repeated measures ANOVA showed that bupropion did not produce a significant effect on time spent eating the sucrose pellets (F(3,39) = 2.38, *p* = 0.08), or time sniffing the neutral odor (F(3,39) = 0.45, *p* = 0.71) (Fig. 2A and C). However, bupropion significantly increased time running in the RW (F(3,39) = 3.26, *p* < 0.05). Planned comparisons revealed that all doses of bupropion (5.0, 10.0, and 15.0 mg/kg) significantly increased time running in the RW compared to the vehicle group (*p* < 0.05 for the two lower doses and *p* < 0.01 for the highest one; Fig. 2B). Moreover, repeated measures ANOVA also showed a significant effect of bupropion on time spent in the RW compartment (F(3,39) = 4.35, *p* < 0.01), and time spent in the food compartment (F(3,39) = 3.92, *p* < 0.01). Planned comparisons showed that all doses of bupropion increased time spent in the RW compartment in comparison with the vehicle group (10.0 mg/kg *p* < 0.05, and 5.0, and 15.0 mg/kg *p* < 0.01)**.** Similarly, bupropion-treated mice spent less time in the sucrose pellet compartment in comparison with the vehicle group (10.0 mg/kg *p* < 0.05, and 5.0, and 15.0 mg/kg *p* < 0.01). However, there was no significant effect of bupropion on time spent in the neutral odor compartment (F(3,39) = 1.78, *p* = 0.16) (Fig. 2E). Finally, bupropion did not produce significant differences in the number of entries into the food compartment (F(3,39) = 1.39, *p* = 0.42), entries to the RW compartment (F(3,39) = 1.39, *p* = 0.25), or entries to the neutral odor compartment (F(3,39) = 0.13, *p* = 0.93) (Fig. 2D).

### Effect of bupropion in combination with TBZ on preference for reinforcers as measured in the 3-choice-T-maze task

**Fig. 3:**
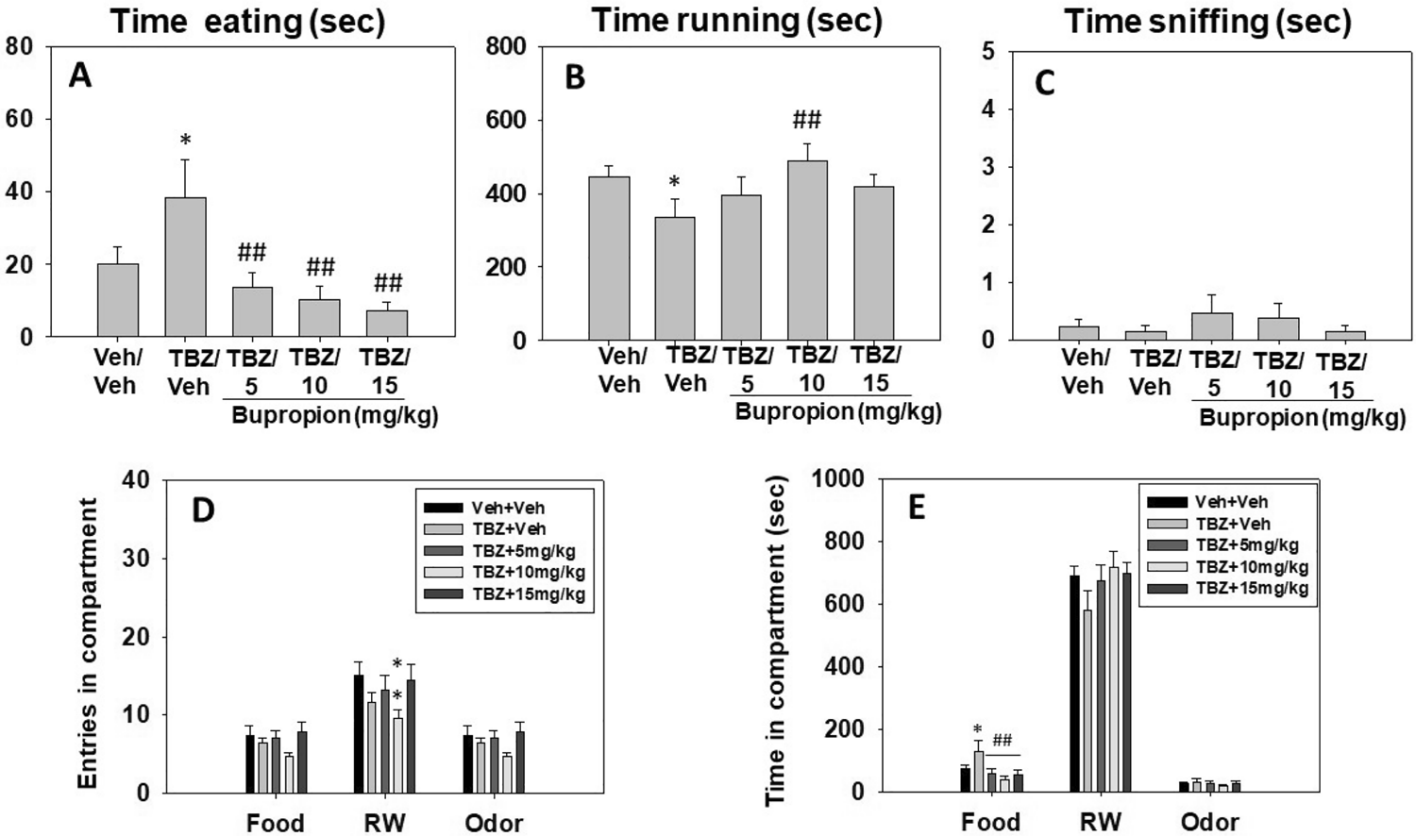
Effect of TBZ (vehicle or 8 mg/kg) plus bupropion (vehicle, 5, 10, and 15 mg/kg) combination on time eating (**A**), time running (**B**), time sniffing (**C**), entries into compartments (**D**), and time spent in each compartment (**E**) of the T-maze task assessed during 15 min. Bars represent mean ± S.E.M. of accumulated seconds or number of entries. *p < 0.05, **p < 0.01 significantly different from Veh-Veh. ##*p* < 0.01 significantly different from TBZ-Veh

A new group of mice (*N* = 13) received TBZ (8.0 mg/kg) and 90 min later (30 min before being placed in the T-maze during 15 min), they received a dose of BUP (5.0, 10.0, and 15.0 mg/kg, randomly varied order, repeated measures design). Thus, animals received one drug combination (Veh-Veh, TBZ-Veh, TBZ-BUP 5.0, TBZ-BUP 10.0, and TBZ-BUP 15.0 mg/kg) every week during 5 weeks in a random varied order. Repeated measures ANOVA resulted in a significant effect of treatment on time spent eating the sucrose pellets (F(4,48) = 4.60, *p* < 0.01). Planned comparisons revealed TBZ-Veh increased the time animals spent eating sucrose pellets (*p* < 0.05) compared to Veh-Veh, and all doses of bupropion were able to decrease significantly time consuming the food (*p* < 0.01) in comparison with the TBZ-Veh condition (*p* < 0.01) (Fig. 3A). In addition, repeated measures ANOVA also show a significant effect of treatment on time running in the RW (F(4,48) = 3.06, *p* < 0.05). Planned comparisons revealed that the TBZ-Veh condition significantly reduced time running in the RW (*p* < 0.05) in comparison with the Veh-Veh condition. The TBZ-BUP 10.0 mg/kg treatment reversed this effect, increasing time running in the RW when compared to the TBZ-Veh condition (*p* < 0.01) (Fig. 3B). Finally, the repeated measures ANOVA did not show a significant effect on sniffing the neutral odor F(4,48) = 0.54, *p* = 0.70) (Fig. 3C).

Repeated measures ANOVA did not show a significant effect of treatment on time spent in the RW compartment (F(4,48) = 1.93, *p* = 0.11), or time spent in the neutral odor compartment (F(4,48) = 0.91, *p* = 0.46) (data are shown in Fig. 3E). However, there was a significant effect on time spent in the food compartment (F(4,48) = 4.04, *p* < 0.01), with planned comparisons showing that TBZ-Veh–treated mice spent more time in the food compartment in comparison with the Veh-Veh group (*p* < 0.05), and all groups treated with TBZ plus bupropion (5.0, 10.0, and 15.0 mg/kg) reversed this effect when compared with the TBZ-Veh treatment (*p* < 0.01)**.** Finally, repeated measures ANOVA for the dependent variable entries in compartment did not show a significant effect of treatment on total number of entries to the food compartment (F(4,48) = 1.48, *p* = 0.22), or to the neutral odor compartment (F(4,48) = 2.48, *p* = 0.06) (Fig. 2D). However, there was a significant effect on total entries to the RW compartment (F(4,48) = 2.92, *p* < 0.05), and planned comparisons showed that TBZ-BUP 10.0 mg/kg treated mice decreased number of entries to the RW compartment in comparison with the Veh-Veh treatment (*p* < 0.01).

### Effect of bupropion on FST performance

**Fig. 4:**
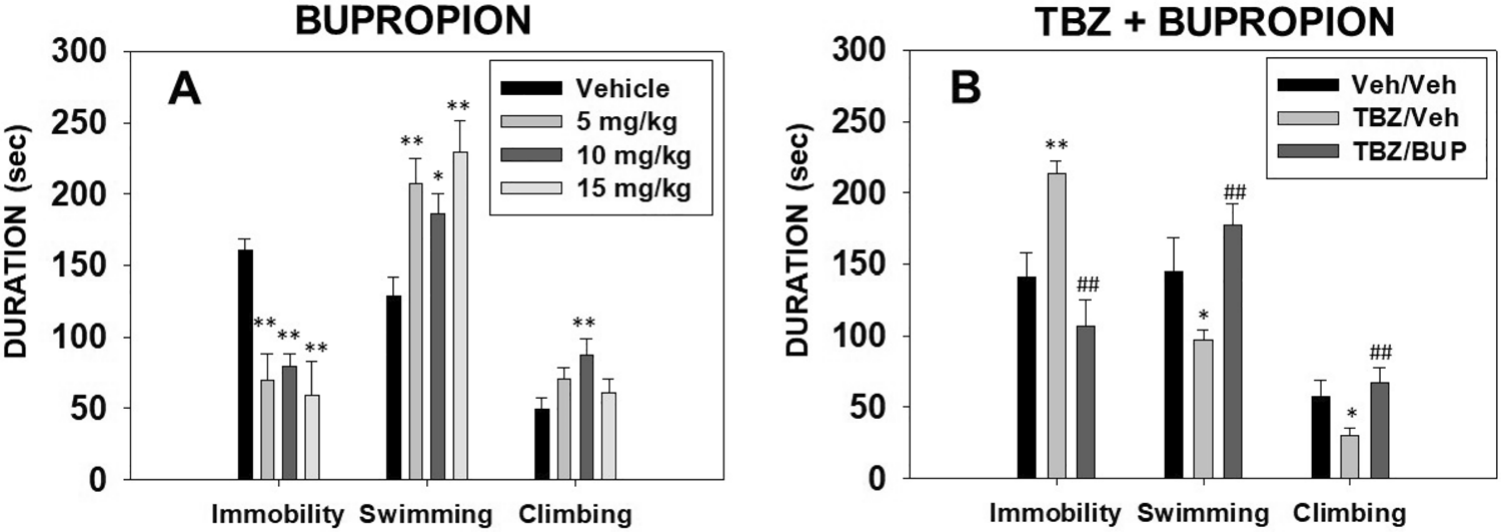
Effect of bupropion (vehicle, 5, 10, or 15 mg/kg) (**A**) and TBZ plus bupropion combination (Veh-Veh, TBZ-Veh, or TBZ-BUP 10 mg/kg) (**B**) on duration of immobility, swimming, and climbing behavior in the FST assessed during 6 min. Bars represent mean ± S.E.M. of accumulated seconds. *p < 0.05, **p < 0.01 significantly different from vehicle or Veh-Veh. ##*p* < 0.01 significantly different from TBZ-Veh

Naïve animals (*N*=40) received vehicle or one dose of BUP (5.0, 10.0 or 15.0 mg/kg) and 30 min after the injection were placed in the FST during 6 minutes. Mice were exposed only once to the FST since behavioral habituation develops in one session. The one-way ANOVA for the different dependent variables showed a significant effect on time spent immobile (F(3,36) = 7.27, *p* < 0.01), time spent swimming (F(3,36) = 5.47, *p* < 0.01), and time spent climbing (F(3,36) = 2.77, *p* < 0.05) in the FST. Planned comparisons revealed that all the groups that received bupropion (5.0, 10.0, and 15.0 mg/kg) displayed significantly less time immobile (*p* < 0.01), and spent more time swimming (*p* < 0.05 for 10.0 mg/kg and *p* < 0.01 for 5.0 and 15.0 mg/kg) in comparison with the vehicle group. However, only the group that received 10.0 mg/kg of bupropion displayed more time climbing than the vehicle group (*p* < 0.01) (Fig. 4A).

### Effect of bupropion in combination with TBZ on FST performance

Naïve mice (*N* = 35) received Veh-Veh or TBZ (8.0 mg/kg)-Veh or Veh-BUP (10.0 mg/kg).

A series of one-way ANOVA showed a significant effect of treatment on immobility (F(2,32) = 13.98, *p* < 0.01), swimming (F(2,32) = 7.65, *p* < 0.01), and climbing (F(2,32) = 4.56, *p* < 0.01). Planned comparisons revealed that TBZ-Veh–treated mice spent more time immobile (*p* < 0.01) and less time swimming and climbing (p < 0.05) in comparison with the Veh-Veh group. Moreover, bupropion was able to reverse these effects, and mice that received the combination of TBZ (8.0 mg/kg) plus bupropion (10.0 mg/kg) spent less time immobile and increased time swimming and climbing (all *p* < 0.01) in comparison with TBZ-Veh treated mice (Fig. 4B).

### Effect of bupropion on anxiety parameters as measured in the DL and EPM paradigms

Mice previously tested in experiment 3.3 (*N* = 40) received 1 week later a second dose of the same treatment (vehicle or BUP 5.0, 10.0, or 15.0 mg/kg), and 30 min after injection, they were placed in the DL box for 5 min. Immediately after this test, they were placed in the EPM for 5 more minutes. Mice were exposed only once to both paradigms, since behavioral habituation develops in one session. The one-way ANOVA for the different dependent variables in the anxiety paradigms did not show significant effects of bupropion on time spent in the illuminated arena (F(3,37) = 0.74, *p* = 0.53) or total of crosses between the lit chamber and the dark chamber (F(3,37) = 0.27, *p* = 0.84) of the DL box (Table 1). Moreover, although the low dose of bupropion tended to reduce time in the open arms of the EPM paradigm, the overall ANOVA did not reached statistical significance (F(3,33) = 2.34, *p* = 0.09). However, there was a significant effect on total number of entries to all arms (F(3,33) = 6.23, *p* < 0.01) in the EPM. Planned comparisons showed that the highest dose of bupropion (15.0 mg/kg) increased significantly total of entries to all arms of the EPM (*p* < 0.01) in comparison with the vehicle group.

**Table 1:** Effect of bupropion on mice behavior in the dark and light box (DL) and elevated plus maze (EPM). Mean (± SEM) seconds or counts in 5 min. **p < 0.01 significantly different from vehicle

	Bupropion (mg/kg)			
	Vehicle	5	10	15
DL box				
Time in the lit chamber (sec)	128.8 ± 8.6	117.8 ± 13.1	116.9 ± 10.1	106.3 ± 12.8
Crosses between compartments	27.0 ± 2.3	25.4 ± 2.8	24.8 ± 4.0	28.9 ± 4.5
EPM				
Time in open arms (sec)	83.6 ± 11.8	49.3 ± 10.8	82.8 ± 9.8	60.5 ± 7.4
Entries to all arms	17.6 ± 2.3	13.0 ± 2.2	17.5 ± 2.5	27.0 ± 1.1**

Further correlational analysis between results in the FST and in the DL and EPM indicate that among the vehicle group, there is a positive and statistically significant correlation between time climbing (*r* = 0.6253, *p* < 0.05) on the one hand, and time swimming (*r* = 0.4588, *p* < 0.05) in the FST with time in the lit chamber of the DL box, indicating that those animals less prone to give up escaping are also more willing to get expose to anxiogenic stimuli. Accordingly, at the lower dose of BUP (5 mg/kg), there was a significant but negative correlation between immobility time (*r* =  − 0.7490, *p* < 0.05) with time in the open arms suggesting that animals that spent more time in a passive state were animals that spent less time in an anxiogenic situation.

### Effect of bupropion in combination with TBZ on anxiety parameters as measured in the DL and EPM paradigms

Mice previously tested in the FST in experiment 3.4. (*N* = 35) received the same treatment combination of Veh-Veh or TBZ (8.0 mg/kg)-Veh or Veh-BUP (10.0 mg/kg) 1 week later. The one-way ANOVA did not show any significant effect of the treatment on time spent in the lit chamber of the DL box (F(2,28) = 0.35, *p* = 0.70), but the treatment produced a significant effect on the total of crosses between compartments (F(2,28) = 5.57, *p* < 0.01) of the DL box. Planned comparisons revealed that the TBZ-Veh group (*p* < 0.01) and the TBZ-BUP (10.0 mg/kg) group (*p* < 0.05) were significantly different from the Veh-Veh group (Table 2). The one-way ANOVA for the variables evaluated in the EPM showed that the treatment produced no significant effect on time spent in the open arms (F(2,26) = 2.70, *p* = 0.08), although it produced a significant effect on the total number of entries to all arms (F(2,26) = 6.67, *p* < 0.01). Planned comparisons indicated that both groups treated with TBZ showed reduced locomotion, TBZ-Veh (*p* < 0.01) and TBZ-BUP 10.0 mg/kg (p < 0.05) compared to the Veh-Veh group.

**Table 2:** Effect of TBZ and BUP combination on mice behavior in the dark and light (DL) box and elevated plus maze (EPM). Mean (± SEM) seconds or counts in 5 min. *p < 0.05, **p < 0.01 significantly different from Veh-Veh

	Veh-Veh	TBZ-Veh	TBZ-BUP 10 mg/kg
DL box			
Time in the lit chamber (sec)	125.6 ± 6.2	117.1 ± 11.5	111.3 ± 13.8
Crosses between compartments	23.6 ± 1.7	12.6 ± 1.5**	17.2 ± 2.2*
EPM			
Time in open arms (sec)	73.3 ± 17.4	100.5 ± 14.3	61.0 ± 8.7
Entries to all arms	19.0 ± 1.4	10.6 ± 1.6**	13.9 ± 1.3*

The Pearson correlational coefficient analysis demonstrated that there was a significant positive correlation between time climbing (*r* = 0.6154, *p* < 0.05) and time in the lit chamber of the DL box in the group that received the drug combination TBZ-BUP (10 mg/kg).

### Effect of bupropion in combination with TBZ on DARPP-32 phosphorylation patters in Nacb as measured by immunohistochemistry

**Fig. 5:**
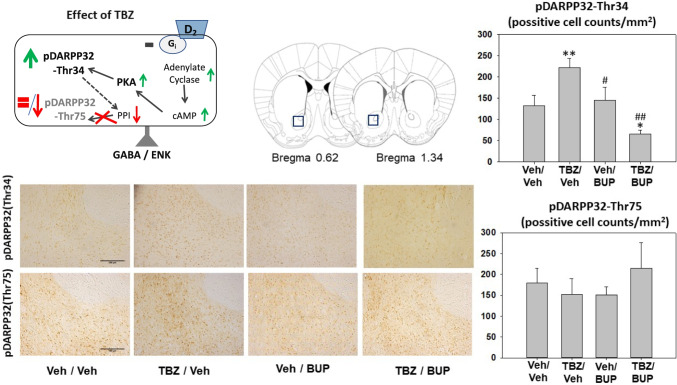
Effect of treatment (Veh-Veh, TBZ-Veh, Veh-BUP, or TBZ-BUP) on number of cell counts positive for pDARPP-32-(Thr34) and pDARPP-32(Thr75) immunoreactivity on Nacb core. Upper left: Diagram showing the effect of TBZ on DARPP-32 phosphorylation in D2-receptor containing cells, and coronal section with bregma coordinates showing location of the brain area for immunohistochemistry taken from Paxino and Franklin’s mouse atlas. TBZ, which depletes DA, increases D2-receptor stimulation by increasing cyclic adenosine monophosphate (c-AMP) production and protein kinase A (PKA) activity yielding to the increase of the dephosphorylation of pDARPP-32(Thr34) and no change or a decrease in the phosphorylation of pDARPP-32(Thr75) by blocking the protein phosphatase (PP1) (based on results from Bateup et al. 2009; Nunes et al. 2013; López-Cruz et al. 2018). Bottom left: photomicrographs of individual representative brain sections. Right: mean ± S.E.M of number of pDARPP-32-(Thr34) and pDARPP-32-(Thr75) counts in a 100um^2^ ROI. *p < 0.05, **p < 0.01 significantly different from Veh-Veh. ##*p* < 0.01 significant differences from TBZ-Veh

Naïve mice (*N* = 21) received Veh-Veh, TBZ-Veh, Veh-BUP 10.0 mg/kg, or TBZ-BUP 10.0 mg/kg 2 h before perfusion. The one-way ANOVA showed an overall effect of treatment on pDARPP-32(Thr34) in Nacb core (F(3,17) = 9.36, *p* < 0.01). Planned comparisons revealed that the administration of TBZ-Veh significantly increased expression of phosphorylated DARPP-32(Thr34) in Nacb core in comparison with the Veh-Veh group (*p* < 0.01). TBZ-BUP treatment reduced pDARPP-32(Thr34) compared to TBZ-Veh (*p* < 0.01) and also compared to Veh-Veh group (*p* < 0.05) (Fig. 5A). The one-way ANOVA showed that there was no significant effect of treatment on pDARPP-32(Thr75) in Nacb core (F(3,17) = 0.52, *p* = 0.66) (Fig. 5B).

### Effect of TBZ on pDARPP-32(Thr75) levels in ventral striatum as measured by Western blot

**Fig. 6:**
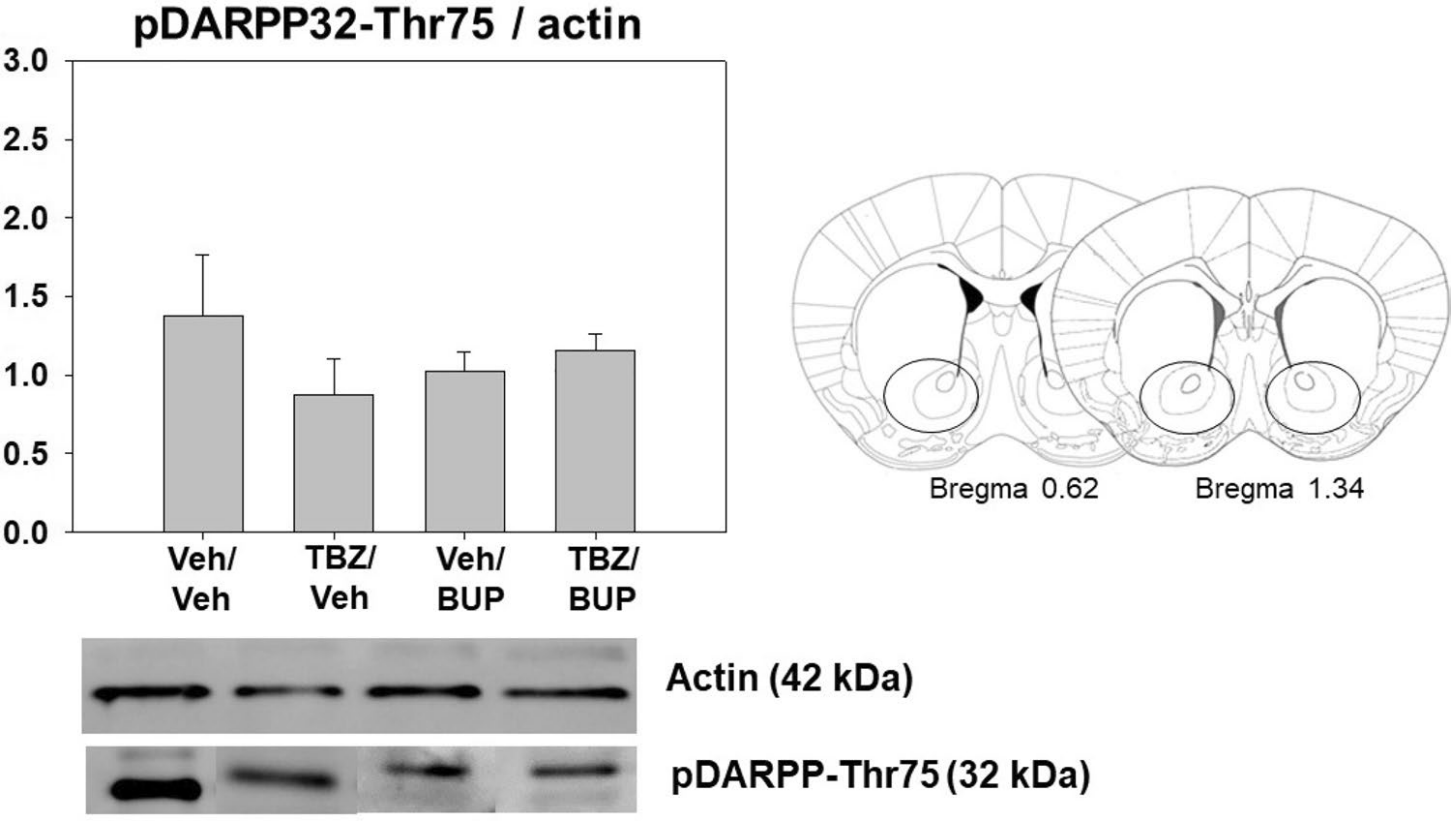
Effect of treatment (Veh-Veh, TBZ-Veh, Veh-BUP, or TBZ-BUP) on pDARPP-32 (Thr75) in ventral striatum. Data are expressed as mean ± S.E.M of density units of pDARPP-32 (Thr75). Lower and right part: representative Western blots showing two bands corresponding to actin (molecular weight of 42 kDa) and pDARPP-32(Thr75) (molecular weight of 32 kDa). Each line contains 30ug of ventral striatum homogenates taken as tissue punctures represented in the coronal brain sections

Naïve mice (*N* = 25) received Veh-Veh, TBZ-Veh, Veh-BUP 10.0 mg/kg, or TBZ-BUP 10.0 mg/kg 120 min before brain extraction. The one-way ANOVA did not show an overall effect of treatment on pDARPP-32(Thr75) when analyzed by Western blotting either (F(3,21) = 0.62, *p* = 0.60) (Fig. 6).

## Discussion

The current studies examined the ability of the catecholamine uptake inhibitor bupropion to produce an effect on selection of reinforcement as assessed in a novel 3-choice-T-maze-task that provides information about the choice between a highly active physical activity (RW) vs. other sources of reinforcement that could be obtained with little effort. Those studies were compared with additional experiments that examined how bupropion was able to modulate immobility and behavioral activation in the FST, a classical paradigm that can evaluate how drugs used as antidepressants potentiate behaviors to scape a stressful unescapable environment. Moreover, we also assessed if the same pharmacological manipulations have an impact on anxiety-related parameters as measured by performance in the DL box and EPM paradigm. In addition, in order to characterize the ability of bupropion to alleviate fatigue-related behavioral activation impairments, we used the VMAT-2 inhibitor TBZ (8.0 mg/kg), since this drug has been reported to induce motivational symptoms such as anergia or fatigue in people (Frank 2009; Guay 2010; Chen et al. 2012), as well as behavioral activation impairments in classical animal models such as the FST or the tail suspension test (Frank 2009; Wang et al. 2010; Carratalá-Ros et al. 2020, 2021), and in effort-related decision-making tasks (López-Cruz et al. 2018; Nunes et al. 2013; Randall et al. 2014; Rotolo et al. 2020, 2019; Yang et al. 2020b; Yohn et al. 2016b; Carratalá-Ros et al. 2020, 2021).

Bupropion is a catecholamine uptake inhibitor that is commonly prescribed as an antidepressant (Dwoskin et al. 2006; Papakostas 2006; Nutt et al. 2007). Bupropion blocks DAT and NET, and as a result of its DAT inhibition, it increases extracellular DA levels and markers of post-synaptic DA-related signal transduction (Hudson et al. 2012; Randall et al. 2015). In humans, bupropion is able to partially alleviate motivational symptoms such as anergia, fatigue, and psychomotor retardation commonly seen in depression (Rampello et al. 1991; Stahl et al. 2004; Demyttenaere et al. 2005; Pae et al. 2007).

In our experiments, mice were tested on the T-maze task, a paradigm that allows animals to freely choose between 3 different non-aversive activities. With this task, animals can choose between a highly preferred reinforcer that requires a high degree of vigor (running in a RW), approaching and consuming a palatable reinforcer (50% sucrose pellets) that is also preferred in contrast to other types of foods (Nunes et al. 2013), or doing a more sedentary activity (sniffing a floral odor (López-Cruz et al. 2018; Carratalá-Ros et al. 2020). Under basal conditions, mice usually spent most of the time engaged in the preferred reinforced activity (the RW), secondly, spent their time eating the sucrose pellets, and the least preferred reinforcer was consistently the non-social odor (López-Cruz et al. 2018; Carratalá-Ros et al. 2020). In the present studies, although the baseline preference for the RW was already high, administration of bupropion was able to significantly increase time spent running in comparison with the vehicle group, and also increased time spent in the RW compartment. This effect was paralleled by a mild reduction in preference for sucrose that was significant in terms of time spent in the food compartment (Fig. 2E), but there was no impact on the least preferred reinforcer (the odor). These doses of bupropion did not seem to affect ambulation in the T-maze as there was no change in number of crosses between compartments.

Our results are in accordance with previous studies in which the administration of different doses of bupropion in rats shifted choice behavior, increasing the tendency to work by lever pressing for food in a highly demanding task (i.e., progressive ratio (Bruijnzeel and Arkou 2003; Randall et al. 2015). Bupropion-treated rats increased work output in order to obtain preferred high carbohydrate food pellets, and they decreased the consumption of concurrently available chow that is much less preferred (Randall et al. 2015). However, in order to improve lever pressing performance, bupropion had to be administered at higher doses (20 and 40 mg/kg; Randall et al. 2015) than the ones used in the present studies. It is possible that higher doses of bupropion alone would have also increase time in the RW to a higher degree showing a sharper dose response curve, but RW time is very high under basal conditions, and our bupropion doses (5–15 mg/kg) already elevated performance to 600 s out of 900 s that is the total duration of the test.

Other more selective DAT inhibitors such as GBR12909, PRX-14040, lisdexamfetamine, MRZ-9547, S-CE-123, S-CE-158, and CT-005404 also produced a significant increase in lever pressing in rats tested on PROG/chow choice tasks, and decreased intake of freely available but less preferred chow (Sommer et al. 2014; Yohn et al. 2016a, b, d, e; Rotolo et al. 2019, 2020). In addition, DAT knockdown mice also were shown to increase the tendency to work for a food reward in a concurrent choice task (Cagniard et al. 2006).

In our studies, bupropion also showed activating effects in mice tested under aversive conditions, decreasing passive behaviors and increasing active behaviors such as swimming and climbing tested in the FST. Previous studies have reported similar effects of bupropion in mice and rats tested on traditional immobility measures in paradigms such as the tail suspension test and the FST (Yamada et al. 2004; Bourin et al. 2005; Kitamura et al. 2008, 2010; Hufgard et al. 2017). However, climbing (or struggling to do so in order to scape a stressful and unescapable situation) has been assessed in relatively few studies involving bupropion (Rénéric and Lucki 1998; Hayashi et al. 2011). Consistent with the present results, in those studies, bupropion increased the time that mice (Hayashi et al. 2011) and rats (Rénéric and Lucki 1998) spent climbing in the FST but at higher doses (30–60 mg/kg) than the ones used in the present results. The maintenance of vigorous and persistent active responding (Gil and Armario 1998) in order to escape is related to DA transmission, and studies that use DA antagonists or DA reuptake inhibitors show a decrease or increase respectively on the output of active behaviors in the FST (Yamada et al. 2004; Kitamura et al. 2010; Li et al. 2015).

Previous studies from our laboratory have demonstrated the anergia-inducing effects of the DA-depleting agent TBZ in effort-based decision-making tasks such as the present T-maze RW-choice task (López-Cruz et al. 2018; Carratalá-Ros et al. 2020, 2021), the T-maze barrier-choice task (Yohn et al. 2015a, b; Correa et al. 2018), and also in operant procedures with different ratio requirements (PROG/chow tasks and FR/chow; Nunes et al. 2013; Randall et al. 2014; Rotolo et al. 2020, 2019; Yohn et al. 2016b). Those effects of TBZ were not due to changes in appetite, food preference, or food reactivity, and did not mimic the effects of reinforcer devaluation (Carratalá-Ros et al. 2020; Correa et al. 2018; Nunes et al. 2013; Pardo et al. 2015; Randall et al. 2014; Yang et al. 2020b). In the present studies, we used a dose of TBZ (8.0 mg/kg) that has been shown to be effective in mice for depleting ventral striatal DA (López-Cruz et al. 2018) and shifting behavior towards sedentary sources of reinforcement (Carratalá-Ros et al. 2020, 2021; Yang et al. 2020b). TBZ-treated mice had significantly reduced time running in the RW and shifted their behavior towards spending more time doing sedentary activities (consuming sucrose pellets) than under the vehicle condition. Bupropion was able to reverse the TBZ-induced bias away from RW activity. In fact, co-administration of 10.0 mg/kg of bupropion to TBZ-treated animals produced a significant reversal of the effects of TBZ, as indicated by a total reversal of the effect of TBZ on time running in the RW, and also decreased time consuming sucrose. In addition, animals exit less times the RW compartment. These results are in accordance with previous studies, in which the administration of these same doses of bupropion to rats alleviated with TBZ-induced anergia in operant procedures such as PROG/chow tasks and FR5/chow tasks (Nunes et al. 2013; Randall et al. 2014), as well as the T-maze barrier-climbing task (Yohn et al. 2015a). In addition, the most effective dose of bupropion (10.0 mg/kg) in the T-maze RW task, and the one that had improved climbing in the FST, also reversed immobility induced by TBZ and restored swimming and climbing to normal levels in TBZ-treated mice. Consistent with the present results, bupropion was able to decrease immobility time in VMAT-2 mutant mice evaluated in the FST (Fukui et al. 2007).

None of these activating effects of bupropion, either when administered alone or in combination with TBZ, seems to be due to anxiolytic actions, since there were no changes in anxiety measures in the DL box or in the EPM (Tables 1 and 2) in the dose range tested. Similar doses of bupropion in adult mice had previously been demonstrated not to have an impact on anxiety-related parameters evaluated in the EPM paradigm (Carrasco et al. 2004, 2013; Redolat et al. 2005a, b). Nevertheless, these doses did have effects on motor activity in mice (Redolat et al. 2005b), and because crossings in the EPM also are sensitive to the locomotor actions of bupropion (Carrasco et al. 2004), we analyzed crosses between compartments in the DL box and entries to all arms in the EPM, as indices of changes in locomotion. Thus, the highest dose of bupropion used in the present experiments (15.0 mg/kg) did increase locomotion in terms of total of entries to all arms in the EPM paradigm, and TBZ decreased total crosses between compartments in the DL box and total of entries to the EPM paradigm. Bupropion at 10.0 mg/kg was not able to reverse this effect in TBZ-treated animals. Thus, none of the doses used in the present experiments seems to modulate anxiety-related behaviors, although some of them can affect locomotion in these paradigms, an effect that was not seen in terms of total number of crosses in the T-maze.

All these results seem consistent with the well know functions of Nacb DA, which has been reported to have a key role in the regulation of voluntary locomotion, response vigor, and effort-related aspects of motivated behavior (Salamone and Correa 2002, 2012). Bupropion, which blocks both DAT and NET, increased extracellular DA levels in Nacb as measured by microdialysis (Randall et al. 2015), and also increased expression of the postsynaptic signal transduction marker DARPP-32, which is phosphorylated at different sites depending on the type of DA receptor that is activated (Svenningsson et al. 2002; Bateup et al. 2009; Randall et al. 2015). In contrast, TBZ reduced both tissue and extracellular DA levels (López-Cruz et al. 2018; Nunes et al. 2013; Yang et al. 2020b), and produced a pattern of phosphorylated DARPP-32 effects that is consistent with a reduction of post-synaptic DA signaling (López-Cruz et al. 2018; Nunes et al. 2013; Randall et al. 2015). In the present studies, TBZ significantly increased pDARPP-32(Thr34) in Nacb core compared to vehicle treated mice, and this effect of TBZ was reversed by bupropion (10.0 mg/kg). Previous studies with rats have shown that low doses of TBZ preferentially reduced levels of striatal DA compared to its effects on NE and 5-HT (Pettibone et al. 1984; Tanra et al. 1995), and that the effects of TBZ on effort-based choice are reversed by selective DAT inhibitors but not by inhibitors of NET or 5-HT transport (Yohn et al. 2016c, e; Rotolo et al. 2019, 2020, 2021). Nevertheless, in the present mouse studies, a possible role for NE cannot be ruled out, and recent research indicates that NE also plays a role in effort-based choice, especially exertion of effort based on force output (Varazzani et al. 2015; Borderies et al. 2020).

In the present studies with mice, TBZ did not affect phosphorylation of DARPP-32 at threonine 75 (pDARPP-32(Thr75)) in Nacb core, using different antibodies and using two different techniques, immunoblotting and immunohistochemistry. These results seem to differ from previous studies using immunohistochemical techniques in rats, in which TBZ increased both phosphorylated forms of DARPP-32 (Thr34 and Thr75) in Nacb core in different groups of neurons containing different type of DA receptors; pDARPP-32 (Thr34) increases in D2 containing neurons, while pDARPP-32 (Thr75) does so in D1 containing neurons (Nunes et al. 2013). Nevertheless, these findings are in line with previous results in mice in which Western blotting methods were used; TBZ only produced an increase on phosphorylation of DARPP-32(Thr34) but not on DARPP-32(Thr75) in ventral striatum (López-Cruz et al. 2018). The effect of TBZ only on DARPP-32(Thr34) and not on DARPP-32(Thr75) could be explained by differences in the distribution pattern of DA receptors in the dorsolateral–ventromedial striatum of mice (Fukuda 2009; Miyamoto et al. 2018). Generally, the increase of DARPP-32(Thr75) is thought to be the result of an increase of D1 receptor stimulation in substance P positive neurons. However, it seems the dorsolateral and ventrolateral domains of the rostral striatum of mice have low proportion of D1 receptors demonstrated by a weak substance-P labeling (Miyamoto et al. 2019). Thus, the possible low proportion of D1 receptors in substance P-containing neurons could explain the lack of effect of the present pharmacological manipulations on phosphorylated DARPP-32 (Thr75). Taken together, these results suggest that there is a substantial action of TBZ on neurons containing D2 receptors (Nunes et al. 2013). Reductions in D2 receptor transmission have been shown to increase pDARRP-32(Thr34) and to decrease or show no change in pDARRP-32(Thr75) (Bateup et al. 2009; Bonito-Oliva et al. 2011; Nunes et al. 2013; Svenningsson et al. 2000). D2 receptors are co-localized on enkephalin-containing medium spiny neurons (MSNs) (Svenningsson et al. 2000; Bateup et al. 2009). In addition, in the present studies, bupropion was able to reverse the induction of pDARPP-32(Thr34) produced by TBZ, suggesting a predominant effect of both drugs on D2 receptors situated in striatal enkephalin-containing MSNs.

In summary, our results demonstrated that bupropion increases the motivation to select highly active alternatives in choice situations, and to maintain activity under different types of behavioral settings (stressful or reinforcing). Together with previous studies using different rodent and behavioral models (Randall et al. 2012, 2014, 2015; Yohn et al. 2015b, 2016b), our results also show how bupropion improves impaired behavior after injection of TBZ, and also increases exertion of physical effort when administered alone (Randall et al. 2012, 2014, 2015). These results are supported by other studies in which bupropion was shown to exert its effects at doses that augment pre- and post-synaptic markers of DA transmission (Nunes et al. 2013; Randall et al. 2015; Yohn et al. 2015b). Moreover, bupropion also has demonstrated to have therapeutic effects on motivational symptoms in humans (Papakostas et al. 2006; Pae et al. 2007; Argyropoulos and Nutt 2013; Soskin et al. 2013), and the rank order of clinical effectiveness in depressed patients with psychomotor retardation paralleled the specificity of antidepressants as dopaminergic agents (Rampello et al. 1991; Brown and Gershon 1993; Treadway and Zald 2011; Treadway and Pizzagalli 2014). These motivational symptoms are not treated well by classical antidepressant drugs such as serotonin uptake inhibitors (Stahl 2002; Fava et al. 2014), which seem more effective in patients affected by anxious depression (Rampello et al. 1995). Although repeated administration of antidepressants is commonly done in clinical practice, the present research used acute administration of bupropion, which is consistent with previous studies in rats (Nunes et al. 2013). In rodent studies, either acute or repeated administration of DA transport inhibitors has been shown to be effective at enhancing selection of high effort instrumental actions (Randall et al. 2015; Yohn et al. 2016c, d, e). Importantly, the use of acute administration of DA transport inhibitors to improve motivational function in rodents is consistent with human research reporting that acute administration of DA transport inhibitors enhanced motivational function in depressed patients, with an onset of action within 2–3 h (Stotz et al. 1999).

This idea of different therapeutic drugs having positive actions for some symptoms, but no effect or even negative actions on other symptoms, is consistent with the research domain criterion (RDoC) approach (Cuthbert and Insel 2013). In this sense, the present results also support the idea that alterations in dopaminergic transmission could contribute to the pathophysiology of motivational impairments in depression and other pathologies (Stahl 2002; Treadway and Zald 2011; Salamone et al. 2015), and they emphasize the recent theoretical approaches that take into consideration the diverse symptoms that patients with depression show, to create a more effective and individualized approach (Cuthbert and Insel 2013), encouraging investigators to use animal models for addressing neurobiological questions rather than as models of specific mental disorders (Molendijk and de Kloet 2021).
